# Nutritional Interventions Improved Rumen Functions and Promoted Compensatory Growth of Growth-Retarded Yaks as Revealed by Integrated Transcripts and Microbiome Analyses

**DOI:** 10.3389/fmicb.2019.00318

**Published:** 2019-02-21

**Authors:** Rui Hu, Huawei Zou, Zhisheng Wang, Binghai Cao, Quanhui Peng, Xiaoping Jing, Yixin Wang, Yaqun Shao, Zhaoxi Pei, Xiangfei Zhang, Bai Xue, Lizhi Wang, Suonan Zhao, Yuqing Zhou, Xiangying Kong

**Affiliations:** ^1^Key Laboratory of Low Carbon Culture and Safety Production in Cattle in Sichuan, Animal Nutrition Institute, Sichuan Agricultural University, Chengdu, China; ^2^State Key Laboratory of Animal Nutrition, College of Animal Science and Technology, China Agricultural University, Beijing, China; ^3^Institute of Animal Genetics and Breeding, College of Animal Science and Technology, Sichuan Agricultural University, Chengdu, China; ^4^Animal Husbandry and Veterinary Institute, Haibei, China

**Keywords:** growth-retarded yaks, compensatory growth, cysteamine hydrochloride, active dry yeast, rumen, transcripts, microflora, association analysis

## Abstract

Growth retardation reduces the incomes of livestock farming. However, effective nutritional interventions to promote compensatory growth and the mechanisms involving digestive tract microbiomes and transcripts have yet to be elucidated. In this study, Qinghai plateau yaks, which frequently suffer from growth retardation due to malnutrition, were used as an experimental model. Young growth-retarded yaks were pastured (GRP), fed basal ration (GRB), fed basal ration addition cysteamine hydrochloride (CSH; GRBC) or active dry yeast (ADY; GRBY). Another group of growth normal yak was pastured as a positive control (GNP). After 60-day nutritional interventions, the results showed that the average daily gain (ADG) of GRB was similar to the level of GNP, and the growth rates of GRBC and GRBY were significantly higher than the level of GNP (*P <* 0.05). Basal rations addition of CSH or ADY either improved the serum biochemical indexes, decreased serum LPS concentration, facilitated ruminal epithelium development and volatile fatty acids (VFA) fermentation of growth-retarded yaks. Comparative transcriptome in rumen epithelium between growth-retarded and normal yaks identified the differentially expressed genes mainly enriched in immune system, digestive system, extracellular matrix and cell adhesion pathways. CSH addition and ADY addition in basal rations upregulated ruminal VFA absorption (*SLC26A3, PAT1, MCT1*) and cell junction (*CLDN1, CDH1, OCLN*) gene expression, and downregulated complement system (*C2, C7*) gene expression in the growth-retarded yaks. 16S rDNA results showed that CSH addition and ADY addition in basal rations increased the rumen beneficial bacterial populations (*Prevotella_1, Butyrivibrio_2, Fibrobacter*) of growth-retarded yaks. The correlation analysis identified that ruminal VFAs and beneficial bacteria abundance were significantly positively correlated with cell junction and VFA absorption gene expressions and negatively correlated with complement system gene expressions on the ruminal epithelium. Therefore, CSH addition and ADY addition in basal rations promoted rumen health and body growth of growth-retarded yaks, of which basal ration addition of ADY had the optimal growth-promoting effects. These results suggested that improving nutrition and probiotics addition is a more effective method to improve growth retardation caused by gastrointestinal function deficiencies.

## Introduction

Yaks (*Bos grunniens*) are an ancient bovine mainly distributed on the Qinghai-Tibet Plateau at high altitudes from 3000 to 5400 m. Yaks have an important ecological niche in the plateau ecosystem and play a crucial role in the lives of local herdsman by providing meat, milk, fur and fuel (feces, as living fuel), but yaks remain semi-domesticated livestock, grazing on plateau grassland with a natural breeding mode and without artificial feeding. The Qinghai-Tibet Plateau climate is sharp frost in the long-term cold season from October to May (average temperature -5∼-15°C). Forage is extremely scarce for yaks because the grass is withered and snow-covered in the cold season. Because of yaks’ seasonal reproduction characteristics (mating during June to October and delivery during May to September of the next year after a 265-day gestation period), most of the gestation and neonatal periods of yaks occur in the cold season. Severe malnutrition in early life (gestation and neonatal periods) may restrain development and growth in the future, which is referred to as developmental programming (Du et al., 2010). Therefore, growth-retarded yaks widely exist on the plateau grassland, and their low body weight, high morbidity and mortality decrease their farming economic efficiency.

Our previous study found that growth-retarded yaks had lower serum somatotropic axis hormone concentrations, rumen weights and skeletal muscle protein deposition compared to normal yaks (Hu et al., 2016). The gastrointestinal tract plays a crucial role in feed digestion, nutrient absorption, immune and endocrine functions and is the basis for body nutrient deposition and healthy growth (Celi et al., 2017; Powell et al., 2017). Intestinal microflora plays a critical role in regulating gastrointestinal homeostasis and functions of the host. Microbial metabolites, particularly propionate and butyrate, are considered as the important modulatory media (Ohland and Jobin, 2015). The rumen is an important digestive organ for ruminants to digest high-fiber forage through microbial fermentation, approximately 65% of overall DM digestion occurs in the rumen. The main microbial metabolites are VFA absorbed on the ruminal epithelium and provide 70∼80% of metabolizable energy for the ruminant (Gozho and Mutsvangwa, 2008) and also modulate the ruminal epithelium functions (Li et al., 2016). In addition, severe malnutrition during the early life of animals and humans damages the gastrointestinal structure, functions and microflora and causes subsequent growth failure (Yambayamba et al., 1996; Kerr et al., 2015; Meyer and Caton, 2016), typically in nutritionally stunted children with IBD living in the developing world (Campbell et al., 2003; Wong et al., 2006). Therefore, we hypothesized that rumen functional deficiencies and microflora dysbiosis may play a dominating role in the growth restriction of yaks.

The compensatory growth is a common phenomenon in various animals and occurs with the improvement of nutrient intake following a period of appropriate nutritional restriction to reach the best growth capacity of animals (Hornick et al., 2000). However, simple nutritional improvement may fail to promote the compensatory growth of animals that underwent excessive malnutrition; therefore, more efficient and safe methods to promote compensatory growth are clearly required. Generally, re-nutrition strategies for nutritionally stunted children occur in two steps: one is providing adequate nutrients for the rapid growth, and second is supplementing with micronutrients, prebiotics and probiotics to repair the physiological impairment (Caballero, 2002; Poinsot et al., 2018). Cysteamine hydrochloride (CSH) is a natural substance produced in the hypothalamus and gastrointestinal tract and facilitate gastrointestinal development by exhausting somatostatin (SS) (Barnett and Hegarty, 2016). ADY, a probiotic, can improve the gastrointestinal microflora, immunity and nutrition digestibility (Che et al., 2017; Crossland et al., 2018). To our knowledge, most studies on the CSH and ADY stimulating growth were focused on the normal healthy animal, the effects of improving nutrition and the addition of CSH or ADY on rumen microflora, epithelium functions and body growth of growth-retarded yaks are unclear.

Therefore, we hypothesized that the growth restriction of yaks was mainly caused by rumen microflora dysbiosis and epithelium functional (nutrient absorption and metabolism) deficiencies and that feeding basal rations with CSH or ADY additions would improve the rumen microbial community and epithelial functions and promote the compensatory growth of growth-retarded yaks. In this study, an integrated method combining microbiome and transcript analysis was used to investigate the interaction between ruminal microflora and the epithelium genes of growth-retarded yaks after different nutritional interventions, to provide insights into the effective nutritional intervention and its potential mechanisms for promoting the compensatory growth of growth-retarded livestock.

## Materials and Methods

### Ethics Statement

The animal experiment was performed according to the Regulation on the Administration of Laboratory Animals (2017 Revision) promulgated by Decree No. 676 of the State Council. The protocol was approved by the Institutional Animal Care and Use Committee in Sichuan Agricultural University, Sichuan, China. The best practice veterinary care has been provided and informed consent has been granted by the Animal Husbandry and Veterinary Institute of Haibei Prefecture, Qinghai, China.

### Experimental Animals and Design

The experiment was performed at the farm (altitude from 3200 to 3500 m) of Animal Husbandry and Veterinary Institute of Haibei Prefecture, Qinghai Province, China, from September to November 2015. Growth retardation is defined as a body weight of yaks that are 2-fold standard deviations (SD) less than the average weight of the yak population with the same breed and age (Xu et al., 1994; Hu et al., 2016). Forty 15-month-old Qinghai plateau yaks with growth retardation (72.7 ± 6.03 kg, mean ± SD) were selected and randomly divided into four groups with 10 yaks in each group as follows: pasturing group (GRP), feeding basal ration group (GRB), feeding basal ration addition of CSH (5 g/day each yak, 30% purity, Walcom, Shanghai, China) (Hu et al., 2016) group (GRBC), and feeding basal ration addition of ADY (0.1% of basal ration, *saccharomyces cerevisiae* ≥ 2.0 × 10^11^ cfu/g, Angel Yeast, Yichang, Hubei, China) (Liu et al., 2014) group (GRBY). Another 10 normal-growth yaks (93.5 ± 5.99 kg, mean ± SD) of the same breed, age and gender were selected and treated similar to the yaks in the GRP group as a positive control (GNP). All yaks were purchased from local herdsman who traditionally graze yaks on native pasture without feed supplements. After the yaks were marked with ear tags and immunized and parasites were expelled, a 15-day transitional period followed by 60 days of nutritional interventions was implemented. The yaks in the GRP and GNP groups were pastured on grassland without supplementary feed and housing. Grass samples were collected from the natural pasture once every month for analyzing the nutrient contents. Samples were clipped from ten 1-m^2^ quadrats which were randomly selected on the natural pasture. Then samples were pooled, dried at 65°C for 48 h and grounded to pass through a 0.42-mm sieve. The macronutrient levels of the natural pasture were analyzed (Casperson et al., 2018) and showed as follows (air-dry basis): 4.81% crude protein (CP), 2.52% ether extract (EE), 55.01% neutral detergent fiber (NDF), 36.59% acid detergent fiber (ADF), 10.11% ash. Yaks in the GRB, GRBC and GRBY groups were housed in 15 pens according to treatment, with 2 yaks in each pen (3.5 × 8.0 m). Each pen provided daily rations (at 0900 and 1600 h) and water individually. All yaks had access to grass or daily rations and water *ad libitum*. The basal ration was designed according to the Chinese Beef Cattle Raising Standard 2004 (NY/T 815–2004), and the feed ingredients and nutrition levels are shown in Table 1.

**Table 1:** Ingredients and nutrition levels of the basal ration.

Items	% of Dry Matter
Ingredients	
Corn	18.55
Soybean meal	5.60
Wheat bran	5.95
Rapeseed dregs	2.80
Rapeseed oil	0.28
Calcium hydrophosphate	0.20
Calcium carbonate	0.60
Sodium chloride	0.28
Sodium bicarbonate	0.35
Choline chloride	0.04
Premix (trace minerals and vitamins)^a^	0.35
Oaten hay	65.00
In total	100
Nutrition levels	
Dry matter	89.16
Net energy for gain NEg (MJ/kg)	3.28
Crude protein CP	12.35
Ether extract EE	2.74
Crude fiber CF	21.51
Neutral detergent fiber NDF	46.21
Acid detergent fiber ADF	27.55
Calcium Ca	0.57
Phosphorus P	0.38


*^*a*^The premix provided trace minerals and vitamins to the basal ration containing per kilogram of 4400 IU VA, 550 IU VD, 110 IU VE, 0.10 mg Co, 8 mg Cu, 0.50 mg I, 40 mg Mn, 0.20 mg Se, 60 mg Zn, 100 mg Fe.*

### Sample Collection

The feed consumption of yaks in the GRB, GRBC and GRBY groups were recorded in each pen each day, yaks in GRP and GNP groups grazing on the grassland had no forage intake record. All yaks were weighed on day 0 and 60 before the morning feeding. The ADG was calculated from the initial weight and final weight. The feed to gain ratio (F/G) was calculated from the daily ration DM intake and ADG. Jugular blood samples were collected on the day 60 before the morning feeding, then serums were separated and stored at -20°C for glucose, total protein, triglyceride, lipopolysaccharide (LPS) and TNF-α analysis using ELISA kits (Nanjing Jiancheng, Nanjing, Jiangsu, China). Then six yaks in each group, which were close to the group average weight, were slaughtered after fasted 12 h via captive bolt stunning and exsanguinated humanely. The slaughter process was in accord with the national standard Operating Procedures of Cattle Slaughtering (GB/T 19477-2004). The ruminal contents were collected, mixed from ventral, dorsal and caudal areas of the rumen, and then filtered through four-layer nylon cloth. The pH-value of the ruminal fluids was determined immediately using a pH meter (INESA, Shanghai, China). The ruminal digesta samples (solid parts) were stored at -80°C for bacterial community analysis. Ruminal fluid samples were stored at -80°C for VFA (acetate, propionate, and butyrate) concentrations analysis using gas chromatography (Agilent Technologies, Santa Clara, CA, United States) (Mao et al., 2008). The rumen epithelia (approximately 1 g) in the ventral sac were separated from the serosal and muscular layers, rinsed 20 times with cold sterile PBS, minced and packed into 1.5 ml tubes, and then snap frozen in liquid nitrogen and stored at -80°C for RNA-seq and qRT-PCR analysis (Wang et al., 2017). Additionally, rumen epithelia in the ventral and dorsal sac were cut (2 cm × 2 cm) and placed into 4% paraformaldehyde overnight, then tissues were dehydrated and embedded in the paraffin, cut into 3 sections with 5 μm thick and stained with Hematoxylin and Eosin (H&E). The morphological characteristics were recorded using the Image-Pro Plus 6.0 software (Media Cybernetics Inc., Bethesda, MD, United States) (Malhi et al., 2013).

### Comparative Transcriptome of the Ruminal Epithelium Between Growth-Retarded and Normal Yaks

Ruminal epithelium samples of four yaks in GRP and GNP group, respectively, which were close to the average group weight, were analyzed by RNA-seq. Total RNA was extracted by Trizol reagent (Takara, Dalian, Liaoning, China) reference to the instruction, then concentration and purity of total RNA were detected by a Nano-100 micro-spectrophotometer (Thermo Scientific, Waltham, MA, United States). The RIN was examined by Agilent 2100 Bioanalyzer (Agilent Technologies, Santa Clara, CA, United States), and the RIN of samples was higher than 7.0. All eight sequencing libraries were constructed referenced to the standard procedures (Tang et al., 2015), marked each sample with a unique barcode and performed single-end sequencing using the BGISeq-500 platform in the Beijing Genomics Institute (BGI, Shenzhen, Guangdong, China).

After low quality reads were removed from raw reads, clean reads were obtained. Bowtie 2 (Langmead et al., 2009) and HISAT (Kim et al., 2015) were used to map the clean reads to yak gene and genome reference sequences (version 1.0) (Hu et al., 2012), respectively. RSEM software (Li and Dewey, 2011) was used to computed the values of fragments per kilobase of transcript per million fragments mapped (FPKM) to evaluate the gene expression levels. The relationship between samples were analyzed by Hierarchical Clustering and Principal Components Analysis (PCA) using the online resource^1^. NOISeq method was used to screen DEGs between the two groups. Genes with fold changes ≥ 2 and probability (Probability of significant difference) ≥ 0.8 were identified as DEGs (Tarazona et al., 2011). The Gene Ontology (GO) and Kyoto Encyclopedia of Genes and Genomes (KEGG) were used to perform pathway enrichment analysis of DEGs by the online resource^2^, the *P*-value less than 0.05 determined significant difference (Zhang et al., 2017).

### Nutritional Regulated of Rumen Functional Gene Expressions Detected by Real-Time PCR (qRT-PCR)

The selected key gene expression of the significantly enriched pathways in GO and KEGG analysis were detected via qRT-PCR. Six ruminal epithelium samples of each group were analyzed. Amplification primers of the genes were designed using primer 5.0 software. The functions, names and primer information of these genes were provided in Table 2. The cDNA was reverse transcribed from the RNA, which was extracted according to the method in the RNA-seq analysis section, using cDNA Synthesis Kit (Takara, Dalian, Liaoning, China) reference to the instructions. qRT-PCR was performed using the SYBR Green Kit (Takara, Dalian, Liaoning, China) and CFX96 Touch^TM^ Real-Time PCR Detection System (Bio-Rad, Hercules, CA, United States) reference to the instructions. All samples were performed in three duplicates. Relative expression of genes were calculated by using 2^-ΔΔCt^ method with the housekeeping genes ACTB and GAPDH (Livak and Schmittgen, 2001).

**Table 2:** Information of primers for qRT-PCR.

Functions	Genes	GenBank ID	Primer sequence (5′-3′)	Tm (°C)
Complement system	*C2*	XM_005893783.2	F: TGGCGGCTCCAGTAAGAACTCC	62.3
			R: GCAGGCGGAAGAGGTTGATGTG	
	*C3*	XM_005890706.2	F: GCCTGCTGCTGCTGCTTCTAG	64.0
			R: CCACAGTCTCCTCGCTCTCCAG	
	*C7*	XM_005889717.2	F: GTGTGAGCAAGGCGTCCTCTTAG	62.5
			R: CCAGCTCCTCGTCTTCGCATTG	
Ion and VFA absorption	*SLC40A1*	XM_005889537.1	F: AGGCGTCATTGCTGCTAGAATCG	56.0
			R: GGAGCCAGGATGACCATGATGAAG	
	*SLC31A1*	XM_005902484.2	F: ACAGTGCTGCACATCATCCAAGTG	60.4
			R: TCCACTACCACCGCCTTCTTCC	
	*CLCN2*	XM_005909509.2	F: CACAGACGGCAGCACTTACAGG	64.0
			R: CGGCGATCATAACAGGCAGGATG	
	*SLC26A3*	XM_005901422.1	F: CACCACCGCTGCCGCTATTC	54.7
			R: CCACCACCAGGATAACCAAGGATG	
	*PAT1*	XM_005909349.1	F: GGGCACTTCTTCGATGCTTCT	59.5
			R: GTCGTGGACCGAGGCAAA	
	*MCT1*	NM_001037319.1	F: CGGCTCAGATCACCAAGCACAAG	58.0
			R: TTAGGTATCTGCCGAGCGACTCC	
Epithelial cell junction	*CLDN1*	XM_005897671.2	F: GCTGTGGATGTCCTGCGTGTCR	65.0
			R: CCTCGTCGTCTTCCATGCACTTC	
	*CLDN4*	XM_005892850.2	F: TCATCGGCAGCAACATCGTCAC	63.5
			R: CAGCAGCGAGTCGTACACCTTG	
	*OCLN*	XM_005889348.2	F: GCACGTTCGACCAATGCTCT	64.5
			R: CAGGCAAGAGTGGAGGCAAC	
	*ZO1*	XM_014476599.1	F: GAACACGACAGAGCAGCACATAGG	65.0
			R: GGCTCGGAGAGGTGGCTAGTG	
	*CDH1*	XM_005896515.2	F: TGAACACCTACAACGCTGCCATC	63.5
			R: TGCTCAAGCCTTCGCCGTTAAG	
	*DSG2*	XM_014479886.1	F: AGGTGGTGCTGCCTCTTCTGG	63.5
			R: CACGATGGCTTCCTTGGCTGTC	
Housekeeping genes	*ACTB*	XM_005887322.2	F: CAGGTCATCACCATCGGCAA	60.4
			R: TAGAGGTCCTTGCGGATGTCG	
	*GAPDH*	XM_014482068.1	F: CCTGGAGAAACCTGCCAAGTAT	60.4
			R: GAGTGTCGCTGTTGAAGTCGC	


### Microbial DNA Extraction, Library Construction and Sequencing

The rumen digesta samples of four yaks in each group, which were close to group average weight, were detected by 16s rDNA sequencing. Bacterial total genome DNA was extracted by using the TIANamp Stool DNA Kit (Tiangen Biotech. Beijing, China) with the bacterial lysis step, bead-beating step using a TGrinder H24 Tissue Homogenizer (Tiangen Biotech. Beijing, China) and subsequent DNA purification using spin column, as described in the handbook. The concentration and purity of DNA was detected by 1% agarose gels and the Nano-100 micro-spectrophotometer (Thermo Scientific, Waltham, MA, United States). The universal primer (341F: 5′-CCTAYGGGRBGCASCAG-3′ and 806R: 5′-GGACTACNNGGGTATCTAAT-3′) with sequencing adapter and barcode were used to amplified the V3-V4 region of the 16S rRNA gene (Xia et al., 2015) using the Bio-rad T100^TM^ Thermal Cycler (Bio-rad Inc., Hercules, CA, United States). The PCR products were qualified on 2% agarose gel, samples with bright main strip between 400 and 450 bp were chosen for further analysis using GeneJET Gel Extraction Kit (Thermo Scientific, Waltham, MA, United States). Sequencing libraries were generated using NEB Next^®^ Ultra^TM^ DNA Library Prep Kit for Illumina following manufacturer’s recommendations. Then samples were sequenced on an Illumina HiSeq platform (Illumina, San Diego, CA, United States) at Novogene Bioinformatics Technology Co., Ltd. (Tianjing, China).

Paired-end reads from the original DNA fragments were merged by using FLASH and assigned to each sample according to the unique barcodes. Then, reads were filtered by QIIME quality filters (Caporaso et al., 2010). Sequences with ≥ 97% similarity were assigned to the same OTUs. The OTUs contained more than 3 counts in at least one of the samples were retained for the further analysis (Shen et al., 2017). The representative sequences of each OTU were picked to annotate taxonomic information via using the Ribosomal Database Project (RDP) classifier (identity threshold of 0.8). The RDP classifier can accurately provide taxonomic assignments that 98% of the classifications are of high accuracy (98%) and high estimated confidence (≥95%) (Wang et al., 2007). The RDP classifier used the SILVA 128 database, which has taxonomic information predicted down to the species level (Quast et al., 2013; Glöckner et al., 2017). Then OTUs were normalized to the relative abundance in each sample. The alpha diversity included Chao1 and Shannon index were calculated with rarefaction analysis. Principal Coordinate Analysis (PCoA) was analyzed by the online resource^3^.

### Statistical Analyses

The differences of data related to growth performance, serum parameters, rumen morphology, mRNA expressions, rumen fermentations and bacterial abundances between groups were analyzed using One-way ANOVA followed Duncan’s *post hoc* testing in SPSS v.19.0, results were presented as means ± SEM. *P*-values less than 0.05 were regarded as statistical significance. The correlation between rumen VFA concentrations or bacteria abundances and epithelium gene expression was analyzed by using Spearman’s correlation analysis in SPSS v.19.0, *P*-values less than 0.05 and the absolute value of correlation coefficient more than 0.8 were regarded as significant correlation.

### Sequence Data Accession Numbers

The ruminal epithelium RNA-seq data and 16s rRNA gene sequencing data have been deposited in NCBI Sequence Read Archive database with the submission SRP166811 and SRP166982, respectively.

## Results

### Effects of Nutritional Interventions on the Growth Performance of Growth-Retarded Yaks

The initial weights of growth-retarded yaks were significantly lower than the normal yaks (*P* < 0.05). After different nutritional interventions, the final weight of GRBY group was the closest to the normal yaks. The ADG of the GRP group was only 41.3% of the GNP group (*P* < 0.05). Feeding basal ration significantly increased the ADG of growth-retarded yaks (*P* < 0.05) to achieve a similar growth rate of normal yaks (GNP; *P* > 0.05). Feeding basal ration addition of CSH or ADY either significantly promoted the ADG of growth-retarded yaks to exceed the growth rate of normal yaks (GNP) and reduced the feed to gain ratio (F/G; *P* < 0.05). GRBY had the highest ADG and lowest F/G in the nutritional intervention groups (Table 3).

**Table 3:** Effects of nutritional interventions on the growth performance of growth-retarded yaks (*n* = 10).

Items	Groups					SEM	*P*-Value
			
	GRP	GRB	GRBC	GRBY	GNP		
Initial Weight (IW) kg	71.50^b^	73.80^b^	72.90^b^	72.30^b^	93.45^a^	1.479	0.000
Final Weight (FW) kg	77.77^d^	90.95^c^	96.92^cb^	99.84^b^	108.56^a^	1.724	0.002
Average Daily Gain (ADG) kg/d	0.104^d^	0.286^c^	0.401^b^	0.459^a^	0.252^c^	0.019	0.006
Dry Matter Intake (DMI) kg/d	-	2.93	3.04	3.22	-	0.051	0.056
Feed/Gain Ratio F/G	-	10.42^b^	7.63^a^	7.22^a^	-	0.332	0.000


*GRP, growth-retarded yaks pasturing; GRB, growth-retarded yaks feeding basal ration; GRBC, growth-retarded yaks feeding basal ration addition CSH; GRBY, growth-retarded yaks feeding basal ration addition ADY; GNP, growth-normal yaks pasturing. Data with different small letter superscripts within the same row are significantly different (*P* < 0.05). SEM, standard error of the mean. “—” means there is no data, because yaks grazing on grassland had no DMI record.*

### Effects of Nutritional Interventions on the Serum Parameters of Growth-Retarded Yaks

The serum parameters on day 60 showed that growth-retarded yaks (GRP) had significantly lower serum total protein (*P* < 0.05) concentrations and trended toward lower serum glucose (*P* = 0.051) and higher serum LPS (*P* = 0.089) concentrations compared to normal yaks (GNP). Feeding basal ration significantly increased the serum triglyceride and total protein concentrations (*P* < 0.05) and did not decrease the serum LPS concentrations (*P* > 0.05) of growth-retarded yaks. Basal rations addition of CSH or ADY either significantly increased the serum glucose and total protein concentrations compared to the GRP group (*P* < 0.05) and significantly decreased the LPS concentrations compared to the GRB group (*P* < 0.05) (Table 4).

**Table 4:** Effects of nutritional interventions on the serum parameters of growth-retarded yaks (*n* = 10).

Items	Groups					SEM	*P*-Value
			
	GRP	GRB	GRBC	GRBY	GNP		
Glucose (mmol/l)	3.46^b^	4.01^ab^	4.45^a^	4.39^a^	3.92^ab^	0.098	0.001
Triglyceride (mmol/l)	0.17^b^	0.29^a^	0.30^a^	0.31^a^	0.19^b^	0.012	0.000
Total protein (g/l)	61.71^c^	67.13^b^	71.68^ab^	72.40^a^	68.31^ab^	0.960	0.000
LPS (EU/ml)	0.45^ab^	0.52^a^	0.41^b^	0.39^b^	0.37^b^	0.015	0.025
TNF-α (ng/ml)	0.40	0.45	0.40	0.37	0.34	0.014	0.233


*GRP, growth-retarded yaks pasturing; GRB, growth-retarded yaks feeding basal ration; GRBC, growth-retarded yaks feeding basal ration addition CSH; GRBY, growth-retarded yaks feeding basal ration addition ADY; GNP, growth normal yaks pasturing. Data with different small letter superscripts within the same row are significantly different (*P* < 0.05). SEM, standard error of the mean.*

### Effects of Nutritional Interventions on the Rumen Histomorphology of Growth-Retarded Yaks

**FIGURE 1:**
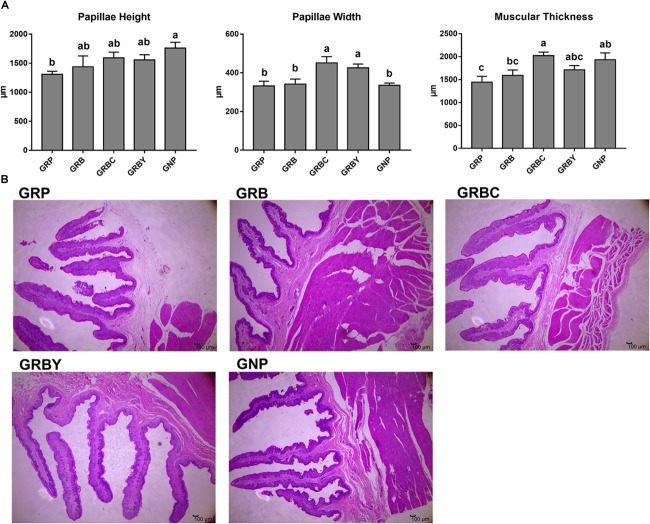
Effects of nutritional interventions on ruminal epithelium histomorphology of growth-retarded yaks. **(A)** Values are means ± SEMs (*n* = 6). Different small letter superscripts represent significantly different (*P* < 0.05). **(B)** Representative micrographs of ruminal epithelium histomorphology, 40× magnification. GRP, growth-retarded yaks pasturing; GRB, growth-retarded yaks feeding basal ration; GRBC, growth-retarded yaks feeding basal ration addition CSH; GRBY, growth-retarded yaks feeding basal ration addition ADY; GNP, growth normal yaks pasturing.

The ruminal papillae height and muscular thickness of the GRP group were significantly lower than the GNP group (*P* < 0.05). Feeding basal ration did not significantly increase the ruminal papillae height and muscular thickness of growth-retarded yaks (*P* > 0.05). Basal rations addition of CSH or ADY either significantly increased the ruminal papillae width (*P* < 0.05). Moreover, basal ration addition of CSH significantly increased the muscular thickness of growth-retarded yaks (*P* < 0.05) (Figure 1).

### Comparative Transcriptome Analysis of the Ruminal Epithelium Between Growth-Retarded and Normal Yaks

The results identified that an average of 13,039 and 12,895 genes per sample in the GRP and GNP groups, respectively. The RNA-seq data information were provided in Supplementary Table 1. The correlation between samples were analyzed by hierarchical clustering (Supplementary Figure 1A) and PCA (Supplementary Figure 1B). In total, 362 genes were significantly differentially expressed in the rumen epithelium between growth-retarded and normal yaks (probability ≥ 0.8, fold change ≥ 2), with 323 genes upregulated and 39 genes downregulated in growth-retarded yaks (Supplementary Figure 1C). The DEGs profiles were shown in the heatmap (Supplementary Figure 1D). The top 20% up- and downregulated (fold-change) DEGs were shown in Supplementary Tables 2, 3, respectively.

**FIGURE 2:**
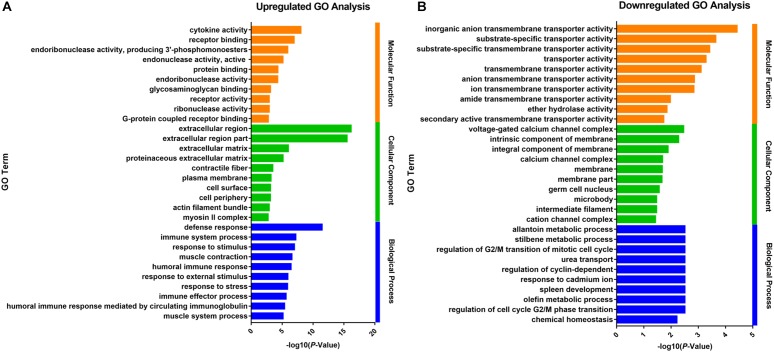
Gene Ontology analysis of the up- **(A)** and downregulated **(B)** DEGs of the ruminal epithelium between growth-retarded and normal yaks. The top 10 GO terms with lowest *P*-values in molecular function, cellular component and biological process were shown, respectively. *Y*-axis represented GO terms and *X*-axis represented the -log10 (*P*-Value).

The up- and downregulated genes were investigated with Gene Ontology (GO) enrichment analysis, respectively (Figure 2). Upregulated genes in growth-retarded yaks were mostly involved in the extracellular region (*P* = 7.15 × 10^-17^), defense response (*P* = 3.02 × 10^-12^), cytokine activity (*P* = 9.59 × 10^-9^), immune system process (*P* = 5.30 × 10^-8^), response to stimulus (*P* = 8.83 × 10^-8^), and extracellular matrix (*P* = 1.03 × 10^-6^), whereas downregulated genes were mostly related to transmembrane transporter activity and intrinsic component of membrane (*P* = 0.005).

**FIGURE 3:**
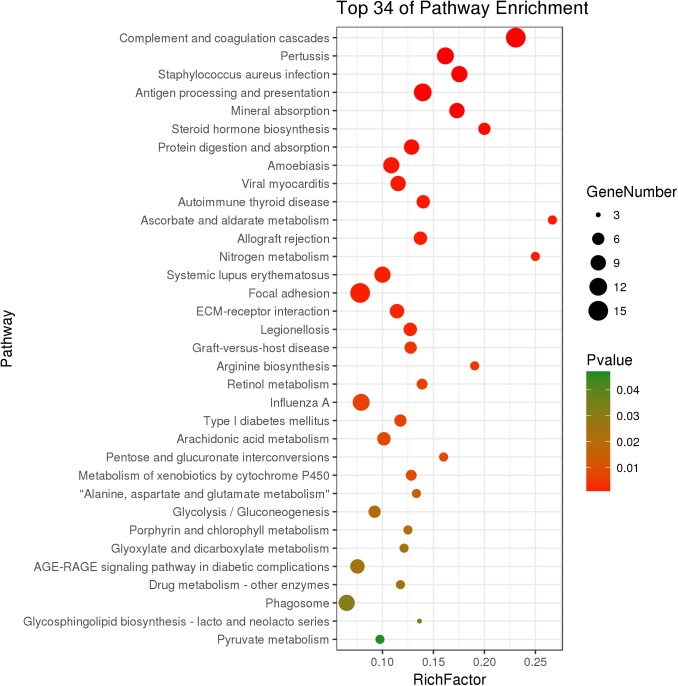
Kyoto Encyclopedia of Genes and Genomes pathway terms (*P* < 0.05) enriched by DEGs of rumen epithelium between growth-retarded and normal yaks. *X*-axis means rich factor (Rich factor = DEGs enriched in the pathway ÷ background genes in the pathway). *Y*-axis represents the KEGG pathway terms. The color of roundness represents *P*-value. The area of roundness represents number of DEGs enriched in this pathway.

The KEGG analysis significantly enriched thirty-four pathways from DEGs (*P* < 0.05) (Figure 3). In the immune system, there were 15 DEGs enriched in the complement and coagulation cascades pathway (*P* = 1.89 × 10^-9^). The genes *C2, C3, C4*, and *C7* had significantly higher expression in growth-retarded yaks compared to normal yaks. The antigen processing and presentation pathway was significantly enriched with 12 genes (*P* = 2.30 × 10^-5^), in which the *MHC I* and *MHC I* genes were typically highly expressed and the *HSPA2* gene was weakly expressed in growth-retarded yaks. In addition, the allograft rejection (7 genes, *P* = 0.001) and phagosome (10 genes, *P* = 0.032) pathways were also significantly enriched. It also identified DEGs enriched in the mineral absorption pathway (9 genes, *P* = 4.40 × 10^-5^). Typically, the expression levels of the nutrient transporter genes including *SLC40A1, HMOX1, SLC31A1, SLC26A3* and *CLCN2* were markedly lower in the ruminal epithelium of growth-retarded yaks compared to normal yaks. There were 15 DEGs enriched in the focal adhesion pathway (*P* = 0.002) and 8 DGEs enriched in the ECM-receptor interaction pathway (*P* = 0.002). The DEGs in the two pathways, including *collagen, fibronectin 1* (*FN1*), *filamin C* (*FLNC*), and *dermatopontin* (*DPT*), had markedly higher expression levels in growth-retarded yaks. Additionally, the cell junction negative regulatory genes *MYL9* and *MYLK* in the focal adhesion pathway had significantly higher expression levels in growth-retarded yaks. In the metabolism functions, the steroid hormone biosynthesis (6 genes, *P* = 3.81 × 10^-4^), metabolism of xenobiotics by cytochrome P450 (5 genes, *P* = 0.009), arachidonic acid metabolism (7 genes, *P* = 0.008), Glycolysis/Gluconeogenesis (6 genes, *P* = 0.020) and pyruvate metabolism (4 genes, *P* = 0.046) pathways were significantly enriched between growth-retarded and normal yaks.

### Effects of Nutritional Interventions on Ruminal Epithelium Functional Gene Expression of Growth-Retarded Yaks

**FIGURE 4:**
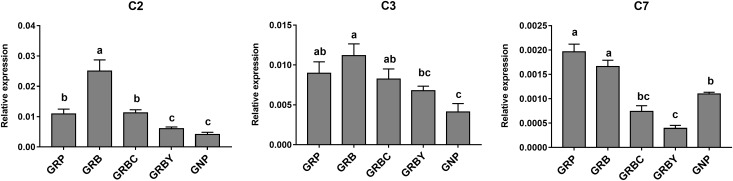
Effects of nutritional interventions on complement gene expressions in the rumen epithelium of growth-retarded yaks. Values are means ± SEMs (*n* = 6). Different small letter superscripts represent significantly different (*P* < 0.05). GRP, growth-retarded yaks pasturing; GRB, growth-retarded yaks feeding basal ration; GRBC, growth-retarded yaks feeding basal ration addition CSH; GRBY, growth-retarded yaks feeding basal ration addition ADY; GNP, growth normal yaks pasturing.

The expressions of representative functional genes related to the significantly enriched pathways (such as complement cascades, mineral transmembrane absorption and epithelial integrity) in GO and KEGG analysis were detected via qRT-PCR. The mRNA expression levels of complement factors *C2, C3* and *C7* in growth-retarded yaks were typically higher than those in normal yaks (*P* < 0.05). Notably, the GRB group had the highest *C2* and *C3* gene expressions in this study. The addition of CSH in basal ration significantly decreased expression of the *C7* gene (*P* < 0.05), whereas the addition of ADY in basal ration significantly decreased *C2* and *C7* gene expression in growth-retarded yaks (*P* < 0.05) (Figure 4).

**FIGURE 5:**
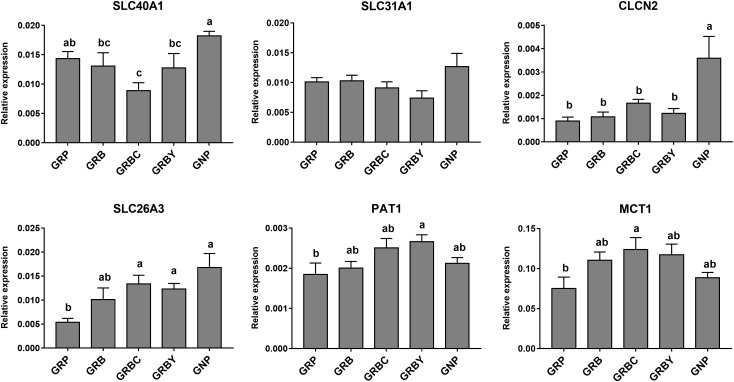
Effects of nutritional interventions on the expression of genes related to ion and VFAs absorption in rumen epithelium of growth-retarded yaks. Values are means ± SEMs (*n* = 6). Different small letter superscripts represent significantly different (*P* < 0.05). GRP, growth-retarded yaks pasturing; GRB, growth-retarded yaks feeding basal ration; GRBC, growth-retarded yaks feeding basal ration addition CSH; GRBY, growth-retarded yaks feeding basal ration addition ADY; GNP, growth normal yaks pasturing.

The nutritional regulations had no acceleration to these gene expressions in growth retardation yaks. The mRNA expression of *SLC26A3* in the GNP groups was nearly 3 times higher than the GRP group (*P* < 0.05). The basal rations addition of CSH or ADY either significantly increased the *SLC26A3* mRNA expression of growth-retarded yaks (*P* < 0.05). The basal ration addition of CSH significantly increased *MCT1* gene expression, and basal ration addition of ADY significantly increased *PAT1* gene expression (*P* < 0.05) compared to the GRP group (Figure 5).

**FIGURE 6:**
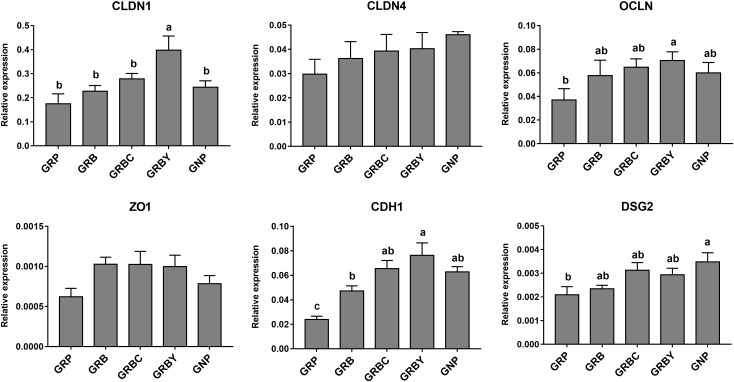
Effects of nutritional interventions on the expression of genes related to barrier function in rumen epithelium of growth-retarded yaks. Values are means ± SEMs (*n* = 6). Different small letter superscripts represent significantly different (*P* < 0.05). GRP, growth-retarded yaks pasturing; GRB, growth-retarded yaks feeding basal ration; GRBC, growth-retarded yaks feeding basal ration addition CSH; GRBY, growth-retarded yaks feeding basal ration addition ADY; GNP, growth normal yaks pasturing.

Growth-retarded yaks had significantly lower *CDH1* and *DSG2* mRNA expression (*P* < 0.05) and trended toward lower *OCLN* mRNA expression (*P* = 0.059) compared to normal-growth yaks. Feeding basal ration (GRB) and CSH addition (GRBC) significantly promoted *CDH1* gene expression (*P* < 0.05), whereas basal ration addition of ADY significantly increased the *CLDN1, OCLN* and *CDH1* gene expression of growth-retarded yaks (*P* < 0.05) (Figure 6).

### Effects of Nutritional Interventions on Rumen Bacterial Fermentation of Growth-Retarded Yaks

Feeding basal ration significantly decreased the rumen pH of growth-retarded yaks (*P* < 0.05), and basal ration addition of ADY effectively alleviated the pH decline induced by basal ration (*P* < 0.05). The GRP group had the lowest ruminal propionate and butyrate concentrations in this study, and feeding basal ration trended toward increasing the butyrate fermentation (*P* = 0.063). The Basal rations addition of CSH or ADY either significantly promoted the propionate and butyrate fermentation compared to GRP group (*P* < 0.05) (Table 5).

**Table 5:** Effects of nutritional interventions on the rumen bacterial fermentation of growth-retarded yaks (*n* = 6).

Items	Groups					SEM	*P*-Value
			
	GRP	GRB	GRBC	GRBY	GNP		
pH	6.93^a^	6.06^c^	6.28^bc^	6.52^b^	6.88^a^	0.066	0.000
Acetate (mmol/l)	33.05	41.52	50.01	47.31	40.22	2.204	0.104
Propionate (mmol/l)	8.29^c^	11.20^abc^	12.86^ab^	13.46^a^	9.05^bc^	0.638	0.034
Butyrate (mmol/l)	4.10^b^	6.27^ab^	6.65^a^	7.80^a^	5.45^ab^	0.395	0.028


*GRP, growth-retarded yaks pasturing; GRB, growth-retarded yaks feeding basal ration; GRBC, growth-retarded yaks feeding basal ration addition CSH; GRBY, growth-retarded yaks feeding basal ration addition ADY; GNP, growth normal yaks pasturing. Data with different small letter superscripts within the same row are significantly different (*P* < 0.05). SEM, standard error of the mean.*

### Effects of Nutritional Interventions on Rumen Bacterial Community of Growth-Retarded Yaks

A total of 1,577,994 raw reads and an average of 65,566 ± 2,231 (SEM) effective reads per sample were identified. A total of 1,974 OTUs were identified. The 16S rDNA sequencing information was provided in the Supplementary Table 4. The results showed that grazing growth-retarded yaks had a significantly higher Chao 1 and lower Shannon index compared to normal yaks (*P* < 0.05). The addition of CSH in basal ration significantly decreased the Chao 1 and Shannon index when compared to the GRP and GNP groups (*P* < 0.05), whereas the addition of ADY in basal ration significantly decreased the Chao 1 when compared to the GRP group (*P* < 0.05) (Supplementary Table 5). The PCOA results showed the distinct microflora among different yak groups (Supplementary Figure 2).

**FIGURE 7:**
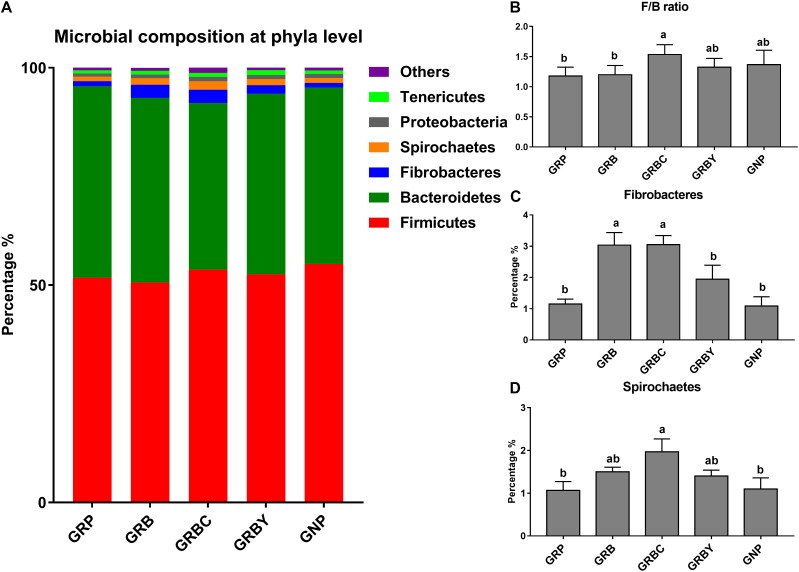
Effects of nutritional interventions on the rumen bacterial composition at phyla level of growth-retarded yaks (*n* = 4). **(A)** Each bar and color represent the average relative abundance of each phyla taxon, the top 6 abundant taxa (relative abundance > 0.5%) were shown. **(B)** showed effects of nutritional interventions on the F/B ratio. **(C,D)** showed effects of nutritional interventions on the relative abundance of Fibrobacteres and Spirochaetes, respectively. Values are means ± SEMs. Bars with different small letter superscripts are significantly different (*P* < 0.05). GRP, growth-retarded yaks pasturing; GRB, growth-retarded yaks feeding basal ration; GRBC, growth-retarded yaks feeding basal ration addition CSH; GRBY, growth-retarded yaks feeding basal ration addition ADY; GNP, growth normal yaks pasturing.

The OTUs annotated 24 bacterial phyla at the phyla level in total. Firmicutes (50.58–54.87%), Bacteroidetes (38.35–44.06%), Fibrobacteres (1.09–3.05%), Spirochaetes (1.07–1.97%), Proteobacteria (0.75–1.01%), and Tenericutes (0.71–1.09%) were the predominant phyla in the yak rumens (relative abundance > 0.5%) (Figure 7A). The GRP group had the lowest ratio of Firmicutes to Bacteroidetes (F/B ratio) in this study. Basal ration addition of CSH significantly increased the F/B ratio (Figure 7B) and increased the abundance of Fibrobacteres (Figure 7C) and Spirochaetes (Figure 7D) in growth-retarded yaks (*P* < 0.05).

**FIGURE 8:**
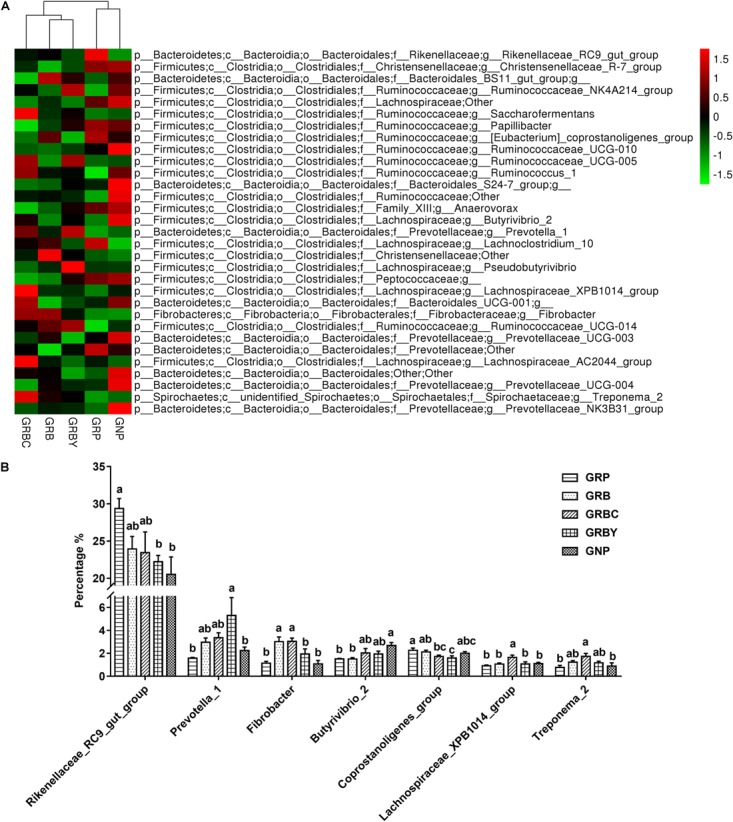
Effects of nutritional interventions on the rumen bacterial composition at genus level of growth-retarded yaks (*n* = 4). **(A)** The heatmap showed the average relative abundance of each dominant genus taxon (relative abundance 0.5%) in different nutritional intervention groups. *Y*-axis represented different bacterial genus and *X*-axis represented different groups. The relative abundance of bacterial genus is represented by color intensity, which is according to the legend. **(B)** The bar graph showed the significantly changed bacteria at genus level. Values are means ± SEMs. Bars with different small letter superscripts are significantly different (*P* < 0.05). GRP, growth-retarded yaks pasturing; GRB, growth-retarded yaks feeding basal ration; GRBC, growth-retarded yaks feeding basal ration addition CSH; GRBY, growth-retarded yaks feeding basal ration addition ADY; GNP, growth normal yaks pasturing.

At the genus level (Figure 8), *Rikenellaceae_RC9_gut_group, Saccharofermentans, Butyrivibrio, Prevotella, Ruminococcus*, and *Fibrobacter* were the dominant genera in yaks (relative abundance 0.5%). The growth-retarded yaks had a higher abundance of *Rikenellaceae_RC9_gut_group* (*P* < 0.05) and a lower abundance of *Butyrivibrio_2* (*P* < 0.05) in the ruminal solid fraction compared to normal yaks. Feeding basal rations significantly increased the abundance of *Fibrobacter* (*P* < 0.05) and trended toward decreasing the abundance of *Rikenellaceae_RC9_gut_group* (*P* = 0.055) in growth-retarded yaks. Basal rations addition of CSH or ADY either significantly decreased the abundance of the *Coprostanoligenes_group* (*P* < 0.05). Moreover, basal ration addition of CSH significantly increased the abundance of *Fibrobacter, Lachnospiraceae_XPB1014_group* and *Treponema_2* (*P* < 0.05) and trended toward increasing the abundance of *Butyrivibrio_2* (*P* = 0.087), whereas basal ration addition of ADY significantly decreased the abundance of the *Rikenellaceae_RC9_gut_group* and increased the abundance of *Prevotella_1* (3.37 times the GRP, *P* < 0.05) in growth-retarded yaks.

### Correlations Between Ruminal Epithelium Gene Expression and Bacteria Populations

**FIGURE 9:**
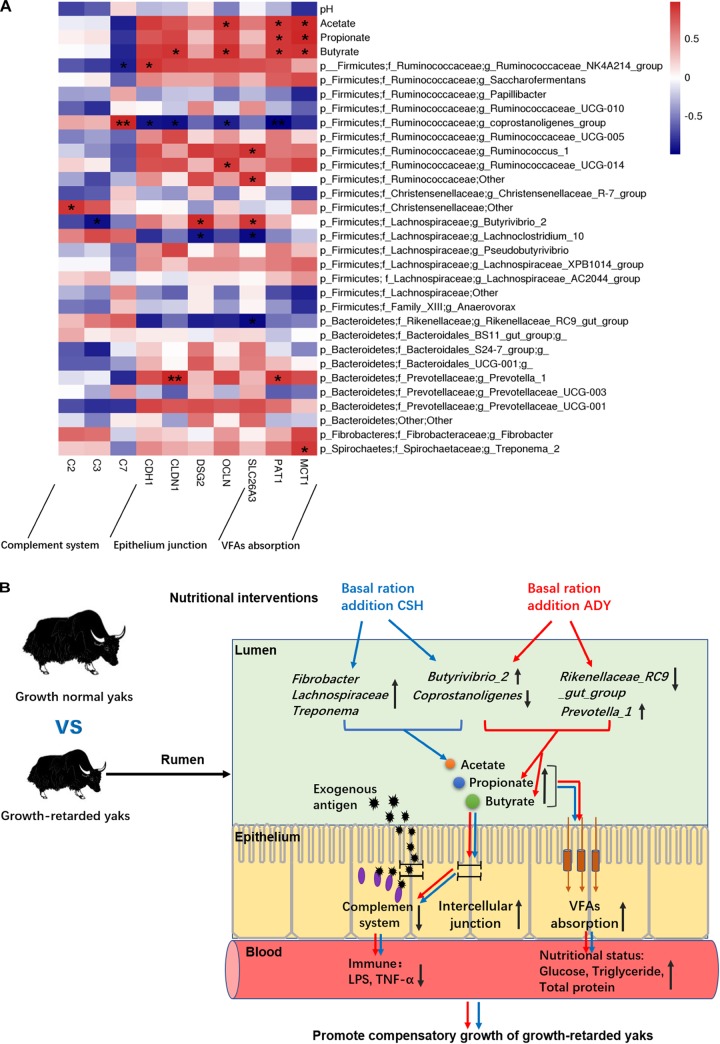
**(A)** The correlations between the ruminal epithelium gene expressions and VFA concentrations or bacteria populations. *Y*-axis represented different VFAs and bacterial genus, *X*-axis represented the different functional genes. Color intensity represents *P*-values of correlation, ^∗^*P* < 0.05 and ^∗∗^*P* < 0.01. **(B)** The proposed model of nutritional interventions promoting the compensatory growth of growth-retarded yaks. The black up and down arrow represent up- and downregulated, respectively. Blue arrows represent the effects of CSH addition, and red arrows represent the effects of ADY addition on the growth-retarded yaks.

The results showed that ruminal microbial product (acetate, propionate and butyrate) concentrations were significantly positively correlated with epithelial VFA absorption gene (*PAT1* and *MCT1*) expressions. Moreover, the acetate concentration was positively correlated with *OLCN* gene expression. The butyrate concentration was positively correlated with epithelial *CLDN1, OLCN* and *CDH1* (*P* = 0.055) gene expressions. The abundance of *Prevotella_1* was positively correlated with *CLDN1* (*P* < 0.01) and *PAT1* gene expression. The *Butyrivibrio_2* population was negatively correlated with *C3* gene expression and positively correlated with *DSG2* and *SLC26A3* gene expressions. Notably, the *coprostanoligenes_group* population was highly positively correlated with *C7* gene expression (*P* < 0.01) and negatively correlated with *CDH1, CLDN1, OCLN*, and *PAT1* (*P* < 0.01) gene expressions. The abundance of the *Rikenellaceae_RC9_gut_group* was negatively correlated with *SLC26A3* gene expression (Figure 9A).

## Discussion

Previous studies investigated the different ruminal epithelium transcriptomes (Kern et al., 2016; Kong et al., 2016) or bacterial communities (McCann et al., 2014; Myer et al., 2015) between high and low growth performance ruminants. To our knowledge, this is the first study to investigate an effective nutritional intervention to promote compensatory growth in growth-retarded yaks with combined transcriptome and microbiome analyses.

### Effects of Nutritional Interventions on Compensatory Growth and Rumen Development of Growth-Retarded Yaks

Under the same breed, age, forage nutrition and environmental conditions, the lower ADG (41.3% of the GNP group), serum glucose and total protein concentrations and ruminal papillae height of the GRP group compared to the GNP group suggested the lower nutrition intake or efficiency of nutrient absorption in growth-retarded yaks. However, feeding basal ration to improve nutrition intake merely increased the ADG, serum glucose and total protein concentrations of growth-retarded yaks to a similar level as the GNP group, although the basal ration had much higher CP and EE contents and lower NDF and ADF contents than those of grass. Furthermore, feeding basal ration did not decrease the serum LPS concentrations. These results indicated that only nutritional improvement cannot significantly improve the ruminal healthy development and nutrient absorption efficiency of growth-retarded yaks. It was well known that improving nutrition intake was helpful for rumen development possibly through improving growth factor secretions or microbial fermentations. High-grain diet significantly affected the mRNA expression of IGF binding protein in the rumen of dairy cattle (Steele et al., 2011). This inconsistent result suggested the potential functional deficiencies existed in the rumen of growth-retarded yaks. The addition of CSH and ADY in basal rations significantly increased the ruminal papillae width and nutritional status (serum glucose and total protein concentrations), decreased the serum LPS concentrations and F/G, and promoted growth rate of growth-retarded yaks to exceed the levels of normal yaks. The results suggested that improving nutrition intake and addition of CSH or ADY may either effectively improve the ruminal epithelium functions in growth-retarded yaks. The CSH and ADY are widely used to promote growth performance of healthy livestock through improving feeds intake and nutrition digestibility. This study suggested that CSH and ADY also had the ability to repair the rumen damage and dysfunction of growth-retarded yaks induced by severe malnutrition.

### Effects of Nutritional Interventions on Ruminal Epithelium Transcripts of Growth-Retarded Yaks

To reveal the potential physiological deficiency and effects of nutritional interventions on the ruminal epithelium of growth-retarded yaks, firstly a comparative transcriptome analysis of the ruminal epithelium between growth-retarded and normal yaks was performed and then the effects of nutritional interventions on functional gene expressions were determined using qPCR.

Gene Ontology analysis results suggested that the physiological dysfunctions of growth-retarded yaks may focus on the inflammatory response, transmembrane transport and cellular junctions of the ruminal epithelium. KEGG results showed that the complement and coagulation cascades pathway was enriched with the lowest *P*-value and *Q*-value, which is an important component of the innate and adaptive immune system (Dunkelberger and Song, 2010) and acts as the first defense of host resistance to potential pathogens (Braem et al., 2015). There are an abundance and diversity of microbes colonized in the rumen. The pathogenic microbes and their metabolites (LPS, glucan) are potential threats to rumen health. The significantly higher expression of complement component genes suggested that the inflammatory response was highly activated in the rumen epithelium of growth-retarded yaks. In addition, the significantly enriched antigen presentation pathway and arachidonic acid metabolism pathway play major role in inflammatory processes in the digestive tract. The steroid hormone biosynthesis and metabolism of xenobiotics by cytochrome P450 pathways significantly enriched in this study mainly work on eliminating dietary byproducts, xenobiotics, and environmental contaminants in cells. These results suggested that the ruminal epithelium of growth-retarded yaks may be invaded by high amounts of heterologous antigens from the lumen.

However, it is surprising that the growth-retarded and normal yaks had significantly different immune responses in the ruminal epithelium when grazing on the same grassland. This phenomenon may be explained by the DEGs including *collagens, filamin* and *fibronectin*, which were significantly enriched in extracellular matrix (ECM) and focal adhesion pathways. These biological macromolecules are complex arrays secreted by cells and distributed in the intercellular space, which contributed to the integrity and barrier function of the ruminal epithelium (Dionissopoulos et al., 2012). Collagen damage or overexpression induces destruction of the integrity and permeability or fibrosis of the epithelium (Steele et al., 2011). In addition, previous studies showed that a high phosphorylation level of MYL catalyzed by MYLK (Jin and Blikslager, 2016) can degrade cell junction complexes (such as tight junctions including CDH1, CLDN1, and OCLN) and increase epithelial barrier permeability (Turner, 2009). The high levels of *MYL9, MYLK, collagens, filamin*, and *fibronectin* gene expressions in our study suggested the rumen epithelial architecture and cell junction deficiency in growth-retarded yaks, which may induce dietary antigens and microbial toxin activation of the complement and antigen presenting pathways.

It was reported that integrated cell junctions were also necessary for nutrient absorption by maintaining the ionic concentration gradients (Wada et al., 2013). Minerals are important components of cells and participate in nutrient metabolism. The GO and KEGG analysis found that growth-retarded yaks had lower gene expression related to ruminal ion transmembrane transport, suggesting that ruminal minerals absorption was blocked. Notably, *SLC26A3* is abundantly located in the rumen epithelium (Xiang et al., 2016) and plays a major role in VFA^-^/ HCO_3_^-^ exchange (Raheja et al., 2010). Beef steers with high feed utilization efficiency had higher *SLC26A3* gene expression on the ruminal epithelium (Kern et al., 2016). The markedly lower expression of *SLC26A3* genes suggested a lower VFA absorption efficiency in the rumen epithelium of growth-retarded yaks.

The representative functional genes related to the complement system, nutrients absorption and epithelial integrity pathways were assessed by using qPCR. The qPCR results were consistent with RNA-seq data showing that immune genes are highly expressed and that cell junction and nutrient absorption genes are weakly expressed in growth-retarded yaks. Unexpectedly, the GRB group had the highest *C2* and *C3* gene expressions, which were activated by exogenous antigen and regarded as the complement components. This may be due to the basal ration (mainly contains non-structural carbohydrates), decreased ruminal pH (Table 5) and increased bacterial fermentation toxins. Concentrate to forage ratio of the basal rations was 35:65, which was widely used in the ruminant farming, but it may be not beneficial for the growth of yaks that had rumen barrier deficiencies. The ruminal epithelium plays a crucial role in response to highly fermentable dietary feed through VFA absorption and barrier function for preventing toxicity (Aschenbach et al., 2011). We identified that simply improving nutritional intake had little benefit for the rumen epithelium barrier repair in growth-retarded yaks, therefore, more toxicants (LPS) may permeate through the ruminal epithelium barrier (Table 4). The addition of ADY in basal ration significantly promoted *CDH1, CLDN1*, and *OCLN* gene expression, exhibiting a more effective for repairing the rumen epithelial barrier in growth-retarded yaks than CSH addition. Studies of monogastric animals showed probiotic bacteria can repair the tight junctions disruption of the intestinal epithelium (Czerucka et al., 2000) Contrary to our study, *Saccharomyces cerevisiae* supplementation had no signification effects on the gene expression of rumen epithelial barrier during weaning in Holstein calves (Fomenky et al., 2018). The possible reasons may be the calves had immature ruminal microflora or functions. Previous study also reported live yeast supplementation during the perinatal period of dairy cow increased the gene expression of rumen epithelial barrier, such as OCLN (Bach et al., 2018). Because of improving the epithelial barrier, basal ration addition of ADY had the optimal effects to decrease the expression of *C2, C3* and *C7* genes in growth-retarded yaks.

### Effects of Nutritional Interventions on Rumen Bacterial Fermentation and Community of Growth-Retarded Yaks

In this study, the PCoA analysis showed that samples of GRP were distinguished from samples of GNP group, suggesting the inherently different rumen microflora between growth retarded and normal yaks. It also found that the nutritional intervention groups (GRB, GRBC and GRBY) seemed to co-mingled together and separate from the grazing yak groups (GRP and GNP). The gastrointestinal microbial community was mainly regulated by dietary factors. Previous study has reported that dietary factors more importantly affected the rumen microflora than host species and geographical environment (Henderson et al., 2015). Recent study also reported the significantly different rumen microflora between grazing and indoor feeding yaks (Zhou et al., 2017) and speculated these differences were mainly caused by different dietary physical characteristics and macronutrient, especially the protein, non-structural carbohydrates and fiber contents. In our study, the basal ration had greater amounts of protein and non-structural carbohydrates than the grass, mainly from corn and soybean meal, and this may be the primary reason for the different microbial clusters between grazing and nutritional intervention groups. The results showed that basal ration addition of CSH increased the F/B ratio of growth-retarded yaks. Previous study found F/B ratio increase were correlated with a high energy harvest, feed efficiency and growth rate of cattle (Myer et al., 2015). Therefore, the addition of CSH in basal ration had the potential to improve the energy utilization efficiency of growth-retarded yaks.

At the genus level, the results found that the *Rikenellaceae_RC9_gut_group* was the dominant genera in the ruminal solid fraction of yaks, which was also reported as the dominant genus in the feces of lambs (Huang et al., 2018) and the rumen of cattle (Pitta et al., 2010; Asma et al., 2013), but other studies reported *Prevotella* was the most dominant genus in the rumen. The inconsistent results in this study may be attributable to breed and diets of host. The *Rikenellaceae_RC9_gut_group* was shown to degrade structural carbohydrates, and starch or oil addition decreased its abundance in the rumen of cows (Asma et al., 2013). *Prevotella* is a dominant beneficial bacteria species in the rumen involved in protein, peptide, starch, hemicellulose and pectin digestion (McCann et al., 2014). In our study, the basal ration addition of ADY greatly increased the abundance of *Prevotella_1* by 237%, suggesting that *Prevotella_1* potentially played a crucial role in promoting compensatory growth. Other studies also reported the rumen *Prevotella* population was related to the feed efficiency of host (McCann et al., 2014; Myer et al., 2015). A recent study found that the grazing yaks had higher abundance of *Prevotella* in the rumen than the indoor feeding yaks (Zhou et al., 2017). However, the protein content of the summer-season pasture was higher than the indoor feeding diet in that study, whereas the cold-season grass had much lower protein level than the basal ration in our study. Therefore, it indicated that dietary protein content may be the predominant contributor to the rumen *Prevotella* population fluctuation. Previous studies found that *Saccharomyces cerevisiae* reduced the relative abundance of *Prevotella albensis* in the rumen (AlZahal et al., 2014). The inconsistent result in our study suggested the ADY may increase other species of the *Prevotella* genus in the rumen of yaks. Interestingly, Basal rations addition of CSH or ADY either significantly decreased the abundance of *Eubacterium coprostanoligenes*. Study also found that goats fed high-grain diet had the higher intestinal abundance of *Eubacterium coprostanoligenes* compared to the goats fed a hay diet (Liu et al., 2018). This genus population may have a negative relationship with dietary nutrition levels. *Butyrivibrio* is a major butyrate producer in the Lachnospiraceae family (Meehan and Beiko, 2014), and ruminal butyrate was reported positively associated with feed efficiency (Guan et al., 2008). Basal ration addition of CSH increased ruminal *Butyrivibrio_2* populations, suggesting that *Butyrivibrio_2* was closely positively related to yak growth performance. Our study also found that basal ration addition of CSH significantly increased the abundance of *Fibrobacter, Lachnospiraceae_XPB1014_group* and *Treponema_2*. These bacteria mainly play a synergistic role on plant material degradation (Nyonyo et al., 2014). To our knowledge, this is the first study to investigate the effect of CSH on rumen microflora. Therefore, mode of action for CSH selectively increasing the fibrolytic bacterial populations requires further research. These results suggested that nutritional interventions changed the ruminal microbial populations and fermentation characteristics of growth-retarded yaks.

### Correlation Between Ruminal Epithelium Gene Expressions and Bacteria Populations

Studies have demonstrated the interaction between gastrointestinal microbes and host gene expressions. Because of the VFA transport functions, the *PAT1* and *MCT1* gene expression was positively correlated with the VFA concentrations. Moreover, the butyrate concentration was also closely positively related with epithelium junction gene expressions. Previous studies found that butyrate acted as signal molecule to improve tight junctions in the rumen epithelium (Zhang et al., 2018), suggesting that beneficial microbes may improve the ruminal epithelium barrier via the butyrate signaling pathway. It also found that the beneficial bacterial genera including *Prevotella_1, Butyrivibrio_2* and *Ruminococcaceae* were positively correlated with cell junctions and VFA absorption genes and negatively correlated with *C3* gene expressions. Therefore, nutritional interventions may repair the ruminal epithelium functional deficiencies of growth-retarded yaks by increasing the abundance of beneficial bacteria and the VFA concentrations (especially butyrate production) in rumen.

In summary, only improving nutrition promoted the ADG of growth-retarded yaks to achieve a similar growth rate of grazing normal yaks, whereas basal rations addition of CSH or ADY either increased the growth rate of growth-retarded yaks to exceed the levels of normal yaks, of which basal ration addition of ADY had the optimal growth-promoting effects. We proposed a possible mechanism as outlined in Figure 9B. The main contributors to the growth retardation of yaks may include the low VFA fermentations in the rumen lumen and the overactive expression of complement genes, aberrant expression of epithelium barrier genes and low expression of nutrients absorption genes in the ruminal epithelium. Basal ration addition of CSH increased the abundance of ruminal plant fiber degradation bacteria, whereas basal ration addition of ADY increased the abundance of *Prevotella_1* and increased the propionate and butyrate fermentations. Basal rations addition of CSH or ADY either increased the ruminal epithelial VFA absorption gene expressions and improved nutritional status of growth-retarded yaks. Basal ration addition of ADY optimally increased the tight junction gene expressions and decreased the complement gene expressions, suggesting that ADY addition potentially improved the ruminal epithelial barrier to decrease exogenous antigen activating complement system. Future studies are needed to investigate the mechanisms of ADY to improve the ruminal barrier, immune and nutrients absorption functions of rumen. The protein expression levels of these representative genes in the ruminal epithelium also need to be verified. Regardless, the study provided a model for researching growth retardation induced by gastrointestinal function deficiencies, which suggested that improving nutrition and probiotics addition may be an important method to treat the growth-retarded animals.

## Author Contributions

ZW and BC designed the research. RH and HZ performed research, analyzed data, and wrote the paper. QP, BX, and LW contributed analytic tools and analyzed sequencing data. XJ, YW, YS, ZP, and XZ performed animal experiments and analyzed samples. SZ, YZ, and XK guided to use yaks farmed equipment and performed research.

## Conflict of Interest Statement

The authors declare that the research was conducted in the absence of any commercial or financial relationships that could be construed as a potential conflict of interest.

## Footnotes

1 http://www.omicshare.com/

2 http://www.omicshare.com/

3 http://www.omicshare.com/

## Abbreviations

ACTB: β-actin
ADG: average daily gain
ADY: active dry yeast
C2: component 2
C3: component 3
C7: component 7
CDH1: cadherin 1
CLCN2: voltage-sensitive chloride channel 2
CLDN1: claudin 1
CLDN4: claudin 4
CSH: cysteine hydrochloride
DEGs: differentially expressed genes
DM: dry matter
DSG2: desmoglein 2
GAPDH: glyceraldehyde 3 phosphate dehydrogenase
IBD: inflammatory bowel disease
MCT1: monocarboxylate transporter 1
MYLK: myosin light chain kinase
OCLN: occludin
OTUs: operational taxonomic units
PAT1: putative anion transporter 1
RIN: RNA integrity number
SLC26A3: downregulated in adenoma
SLC31A1: copper transporter
SLC40A1: iron-regulated transporter
VFA: volatile fatty acids
ZO1: tight junction protein 1
