# Supplementary material for: Nutritional Interventions Improved Rumen Functions and Promoted Compensatory Growth of Growth-Retarded Yaks as Revealed by Integrated Transcripts and Microbiome Analyses
Source: Front Microbiol. 2019 Feb 21;10:318. doi: 10.3389/fmicb.2019.00318 (PMC6393393; doi:10.3389/fmicb.2019.00318)
Supplement: Supplementary file 1 [file Data_Sheet_1.docx]

Supplementary Material

Nutritional Interventions Improved Rumen Functions and Promoted Compensatory Growth of Growth-Retarded Yaks as Revealed by Integrated Transcripts and Microbiome Analyses

**Rui Hu****^#^, Huawei Zou^#^, Zhisheng Wang^*^, Binghai Cao, Quanhui Peng, Xiaoping Jing, Yixin Wang, Yaqun Shao, Zhaoxi Pei, Xiangfei Zhang, Bai Xue, Lizhi Wang, Suonan Zhao, Yuqing Zhou, Xiangying Kong**

# These authors contributed equally to this work

**^*^Correspondence:**

Dr. Zhisheng Wang

[wangzs67@163.com](mailto:wangzs67@163.com)

## Supplementary Figures


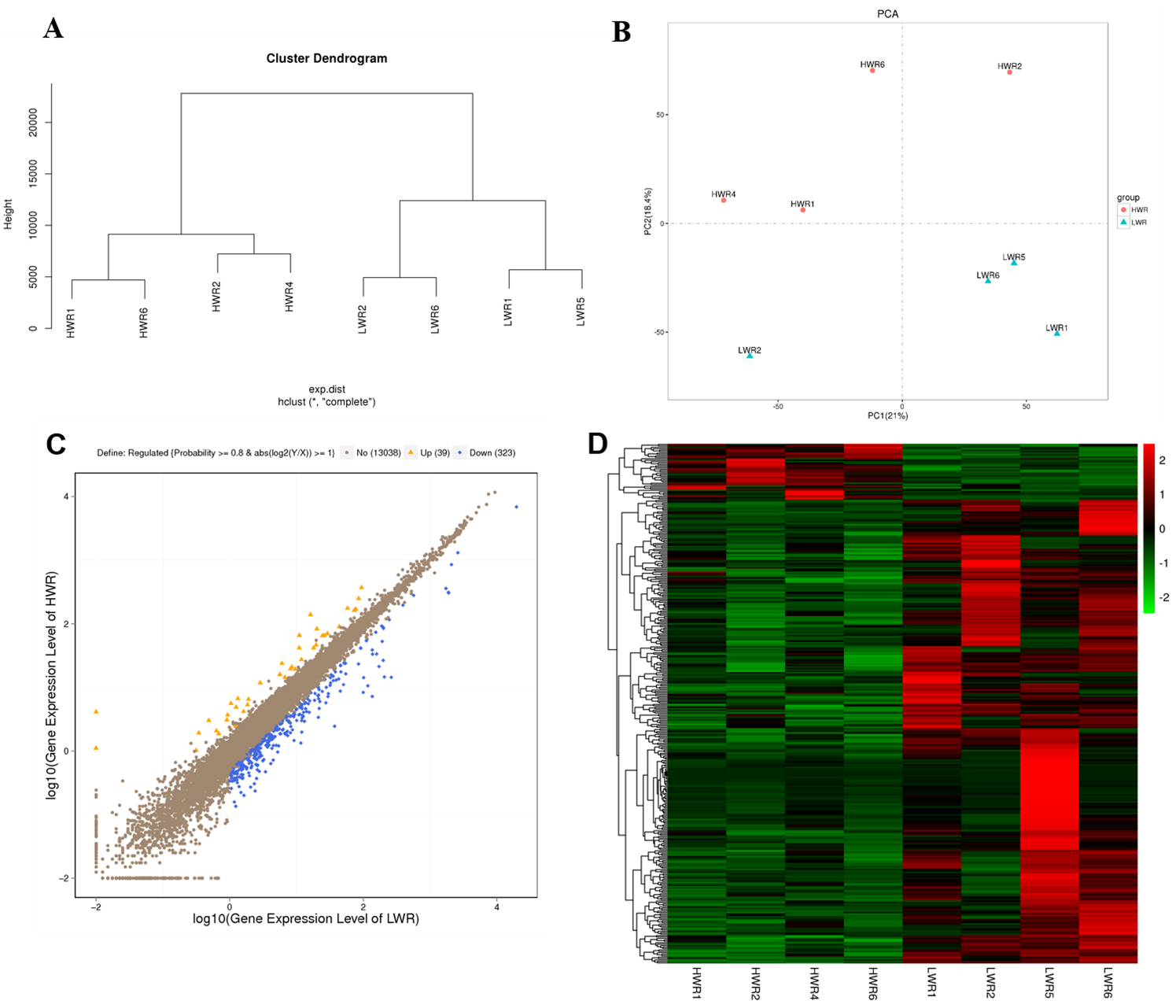


**Supplementary Figure 1.** The different gene expression profiles of the ruminal epithelium between the GRP and GNP groups (n = 4). **(A)** Hierarchical clustering analysis of the GRP and GNP groups. **(B)** PCA plot analysis of the GRP and GNP groups. **(C)** DEGs statistics of the GRP and GNP groups. Blue square represented the significantly upregulated genes in the GRP group; Yellow triangle represented the significantly downregulated genes in the GRP group. **(D)** Heatmap analysis showed DEGs profiling of the GRP and GNP groups. LWR and HWR represent the rumen epithelium samples of the GRP and GNP group, respectively.


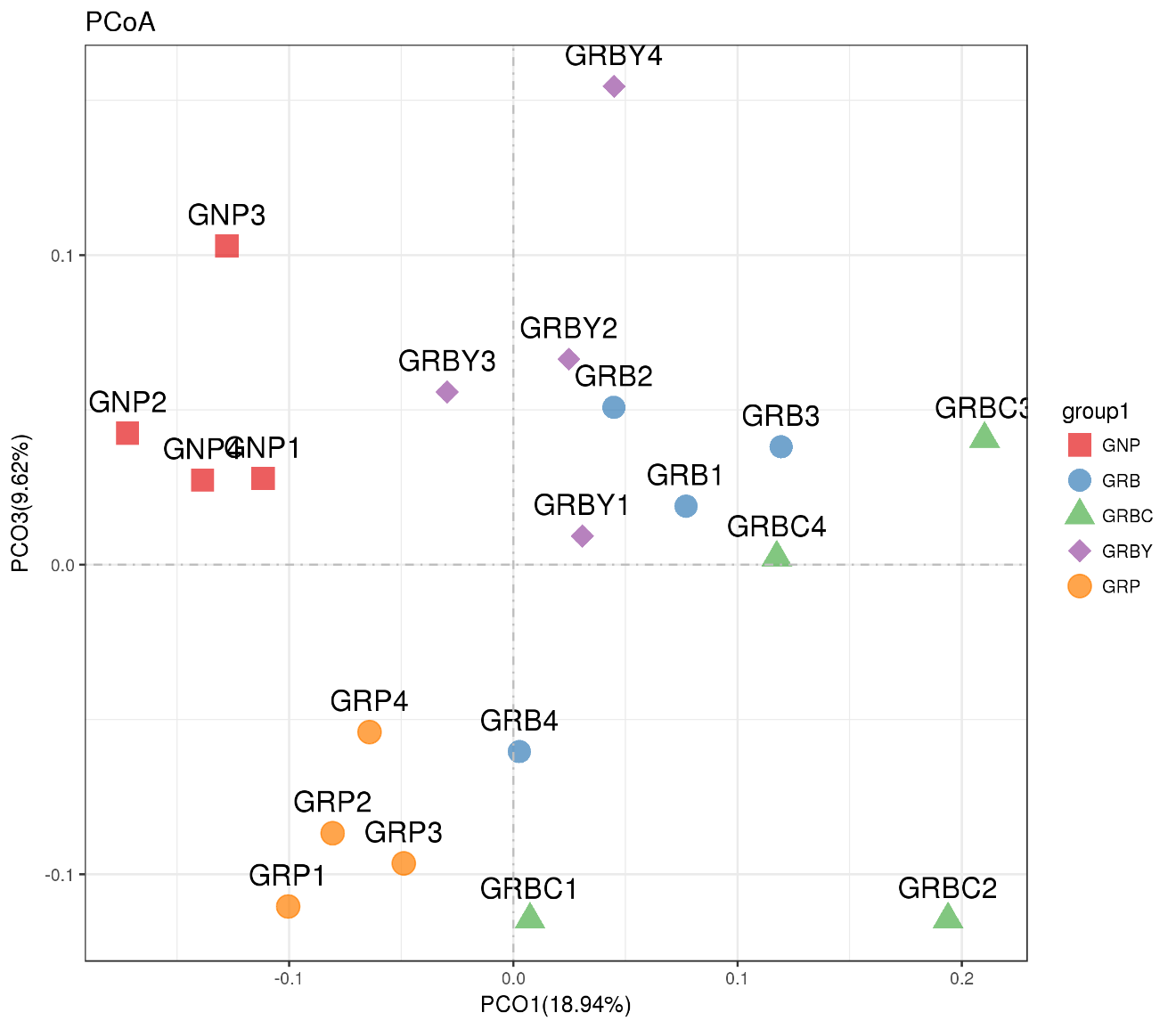


**Supplementary Figure 2.** Effects of nutritional interventions on the microbial PCoA analysis in the rumen of growth-retarded yaks (n = 4). GRP: growth-retarded yaks pasturing. GRB: growth-retarded yaks feeding basal ration, GRBC: growth-retarded yaks feeding basal ration addition CSH. GRBY: growth-retarded yaks feeding basal ration addition ADY. GNP: growth normal yaks pasturing.

## Supplementary Tables

**Supplementary Table 1.** Summary of sequencing data information. Totally, after removed the low-quality reads, 9,307,519,400 bp (base pairs) clean data were produced by 8 ruminal epithelium samples. There were average 23,992,286 clean reads number (clean reads rate 99.59 %) per sample in GRP group, the reads alignment rate to the yak reference genome and gene were 93.68 % and 68.68 %, respectively. There were average 22,545,311 clean reads number (clean reads rate 99.62 %) per sample in GNP group, the reads alignment rate to the yak reference genome and gene were 93.91 % and 67.24 %, respectively.

| Groups | Sample ID | Clean Data Size (bp) | Clean Reads Number | Clean Data Rate (%) | Mapped Reads to genome (%) | Mapped Reads to gene (%) | Identified genes number |
| --- | --- | --- | --- | --- | --- | --- | --- |
| GRP | LWR1 | 1,200,477,600 | 24,009,552 | 99.62 | 93.87 | 68.17 | 13056 |
|  | LWR2 | 1,200,353,150 | 24,007,063 | 99.64 | 93.79 | 69.62 | 13070 |
|  | LWR5 | 1,197,787,050 | 23,955,741 | 99.59 | 93.01 | 68.04 | 13036 |
|  | LWR6 | 1,199,839,350 | 23,996,787 | 99.51 | 94.03 | 68.90 | 12994 |
| GNP | HWR1 | 1,096,586,600 | 21,931,732 | 99.61 | 93.77 | 68.78 | 12978 |
|  | HWR2 | 1,201,449,550 | 24,028,991 | 99.62 | 93.93 | 65.31 | 12842 |
|  | HWR4 | 1,008,555,450 | 20,171,109 | 99.61 | 94.08 | 65.80 | 12952 |
|  | HWR6 | 1,202,470,650 | 24,049,413 | 99.63 | 93.85 | 69.06 | 12807 |

bp: base pairs. GRP: growth-retarded yaks pasturing. GNP: growth normal yaks pasturing. LWR and HWR represent the rumen epithelium samples of the GRP and GNP group, respectively.

**Supplementary Table 2.** The top 20% upregulated DEGs in the ruminal epithelium of growth-retarded yaks compared to normal yaks. (Based on the fold-change)

| Genes ID | GRP average FPKM | GNP average FPKM | log2Ratio (GRP/ GNP) | Probability | genes |
| --- | --- | --- | --- | --- | --- |
| 102283076 | 262.77 | 14.36 | 4.19 | 0.977 | Desmin (DES), partial mRNA |
| 102275623 | 99.89 | 8.33 | 3.58 | 0.956 | Actin, gamma 2, smooth muscle, enteric (ACTG2), transcript variant X3, mRNA |
| 102286924 | 117.08 | 10.59 | 3.47 | 0.961 | Calponin 1, basic, smooth muscle (CNN1), mRNA |
| 102279172 | 2.41 | 0.24 | 3.36 | 0.983 | Haptoglobin (LOC102279172), mRNA |
| 102270358 | 1.23 | 0.14 | 3.19 | 0.960 | Deleted in malignant brain tumors 1 protein-like (LOC102270358), partial mRNA |
| 102283437 | 11.64 | 1.33 | 3.13 | 0.948 | Synemin, intermediate filament protein (SYNM), mRNA |
| 102271687 | 1.64 | 0.20 | 3.03 | 0.960 | Leukocyte immunoglobulin-like receptor, subfamily A (with TM domain), member 6 (LILRA6), partial mRNA |
| 102276577 | 171.94 | 21.27 | 3.02 | 1.000 | Serum amyloid A protein-like (LOC102276577), mRNA |
| 106701159 | 2.34 | 0.31 | 2.94 | 0.943 | WAP four-disulfide core domain protein 3-like (LOC106701159), partial mRNA |
| 102273837 | 2.06 | 0.27 | 2.93 | 0.917 | Kelch-like family member 41 (KLHL41), mRNA |
| 102267504 | 198.71 | 26.55 | 2.90 | 0.974 | Serum amyloid A protein-like (LOC102267504), mRNA |
| 102279611 | 1.17 | 0.16 | 2.90 | 0.947 | Hematopoietic cell signal transducer (HCST), mRNA |
| 102275340 | 101.30 | 14.24 | 2.83 | 0.960 | Tropomyosin 2 (beta) (TPM2), transcript variant X6, mRNA |
| 102286598 | 2.53 | 0.37 | 2.77 | 0.944 | Serine dehydratase (SDS), mRNA |
| 102274376 | 1.48 | 0.22 | 2.73 | 0.923 | Odd-skipped related transciption factor 1 (OSR1), mRNA |
| 102282332 | 4.12 | 0.63 | 2.70 | 0.921 | Sorbin and SH3 domain containing 2 (SORBS2), transcript variant X6, mRNA |
| 102283173 | 9.19 | 1.46 | 2.65 | 0.947 | Ig heavy chain V region PJ14 (LOC102283173), mRNA |
| 102281754 | 21.24 | 3.38 | 2.65 | 0.958 | Class I histocompatibility antigen, Gogo-A*0201 alpha chain-like (LOC102281754), partial mRNA |
| 102287936 | 4.48 | 0.72 | 2.64 | 0.917 | Filamin C, gamma (FLNC), transcript variant X4, mRNA |
| 102267534 | 1896.40 | 305.17 | 2.64 | 0.946 | Peptidase inhibitor 3, skin-derived (PI3), mRNA |
| 102277762 | 11.99 | 1.98 | 2.60 | 0.944 | Ig lambda chain V-II region MGC (LOC102277762), mRNA |
| 102268679 | 2.78 | 0.48 | 2.54 | 0.909 | C-X-C motif chemokine 6 (LOC102268679), mRNA |
| 102282935 | 5.24 | 0.96 | 2.45 | 0.959 | Coiled-coil domain containing 8 (CCDC8), mRNA |
| 102280800 | 45.29 | 8.69 | 2.38 | 0.960 | Immunoglobulin lambda-like polypeptide 5 (LOC102280800), partial mRNA |
| 102267196 | 2.59 | 0.51 | 2.35 | 0.945 | Complement component 7 (C7), mRNA |
| 102278207 | 1.38 | 0.28 | 2.33 | 0.898 | Fatty acid binding protein 7, brain (FABP7), mRNA |
| 106701350 | 1.98 | 0.41 | 2.28 | 0.917 | 60S ribosomal protein L23a (LOC106701350), mRNA |
| 102278927 | 27.57 | 5.70 | 2.27 | 0.966 | Protease, serine, 53 (PRSS53), mRNA |
| 102271680 | 1727.30 | 359.12 | 2.27 | 1.000 | S100 calcium binding protein A8 (S100A8), mRNA |
| 102278717 | 158.62 | 33.45 | 2.25 | 0.994 | Glutathione peroxidase 3 (GPX3), mRNA |
| 102269080 | 21.89 | 4.66 | 2.23 | 0.923 | Myosin light chain kinase (MYLK), transcript variant X10, mRNA |
| 102264614 | 50.62 | 11.02 | 2.20 | 0.920 | Myosin-11 (LOC102264614), mRNA |
| 102270660 | 5.40 | 1.20 | 2.17 | 0.920 | Kininogen 1 (KNG1), transcript variant X4, mRNA |
| 102264496 | 13.15 | 2.93 | 2.17 | 0.921 | Leiomodin 1 (smooth muscle) (LMOD1), mRNA |
| 102280184 | 3.14 | 0.70 | 2.17 | 0.897 | Immunoglobulin iota chain (LOC102280184), mRNA |
| 102277990 | 3.24 | 0.73 | 2.14 | 0.963 | ChaC glutathione-specific gamma-glutamylcyclotransferase 1 (CHAC1), transcript variant X2, mRNA |
| 102275803 | 2.64 | 0.60 | 2.14 | 0.920 | CXADR-like membrane protein (CLMP), mRNA |
| 102279577 | 2.59 | 0.59 | 2.13 | 0.917 | Solute carrier family 6 (amino acid transporter), member 14 (SLC6A14), mRNA |
| 102273141 | 1.93 | 0.45 | 2.09 | 0.896 | Matrix metallopeptidase 9 (MMP9), mRNA |
| 102281952 | 1.57 | 0.37 | 2.09 | 0.894 | Transmembrane protein 61 (TMEM61), mRNA |
| 102279923 | 7.38 | 1.75 | 2.08 | 0.960 | Heparan sulfate (glucosamine) 3-O-sulfotransferase 1 (HS3ST1), mRNA |
| 102270109 | 8.09 | 1.93 | 2.07 | 0.921 | DnaJ homolog subfamily A member 1 pseudogene (LOC102270109), misc_RNA |
| 102275189 | 137.61 | 33.16 | 2.05 | 0.984 | Dual oxidase maturation factor 2 (DUOXA2), mRNA |
| 102270058 | 29.69 | 7.17 | 2.05 | 0.934 | Cldesmon 1 (CALD1), mRNA |
| 102276868 | 1.30 | 0.32 | 2.03 | 0.895 | Calcium channel, voltage-dependent, T type, alpha 1H subunit (CACNA1H), partial mRNA |
| 102280307 | 8.71 | 2.15 | 2.02 | 0.943 | Transmembrane protease, serine 4 (TMPRSS4), mRNA |
| 102267377 | 4.96 | 1.23 | 2.02 | 0.956 | RAB38, member RAS oncogene family (RAB38), mRNA |
| 102276534 | 2.47 | 0.61 | 2.01 | 0.918 | Purkinje cell protein 4 like 1 (PCP4L1), mRNA |
| 102282537 | 1.55 | 0.39 | 2.01 | 0.966 | Protein S (alpha) (PROS1), mRNA |
| 102273400 | 11.94 | 3.01 | 1.99 | 0.918 | MX dynamin-like GTPase 1 (MX1), transcript variant X2, mRNA |
| 102271044 | 1.50 | 0.38 | 1.99 | 0.921 | Nerve growth factor receptor (TNFRSF16) associated protein 1 (NGFRAP1), transcript variant X3, mRNA |
| 102268124 | 4.28 | 1.10 | 1.96 | 0.897 | Chemokine (C-X-C motif) ligand 8 (CXCL8), mRNA |
| 102275017 | 2.09 | 0.54 | 1.95 | 0.916 | Complement C1r-A subcomponent-like (LOC102275017), partial mRNA |
| 102278916 | 15.50 | 4.05 | 1.94 | 0.944 | Chemokine (C-C motif) ligand 19 (CCL19), transcript variant X3, mRNA |
| 102276634 | 30.04 | 7.97 | 1.91 | 0.962 | Ornithine decarboxylase 1 (ODC1), mRNA |
| 102281681 | 1.58 | 0.42 | 1.91 | 0.897 | PDZ domain containing ring finger 3 (PDZRN3), transcript variant X3, mRNA |
| 102279199 | 22.34 | 5.99 | 1.90 | 0.960 | chemokine (C-C motif) ligand 21 (CCL21), mRNA |
| 102270779 | 6.85 | 1.84 | 1.90 | 0.927 | Mucin 1, cell surface associated (MUC1), mRNA |
| 102287458 | 2.14 | 0.58 | 1.89 | 0.916 | Nuclear factor, erythroid 2-like 3 (NFE2L3), mRNA |
| 102264986 | 4.50 | 1.22 | 1.88 | 0.944 | Complement component 1, q subcomponent, B chain (C1QB), mRNA |
| 102271628 | 12.17 | 3.31 | 1.88 | 0.967 | Plasminogen activator, urokinase receptor (PLAUR), mRNA |
| 102267713 | 1.28 | 0.35 | 1.87 | 0.918 | Histone H2B type 1-K (LOC102267713), mRNA |
| 102271231 | 85.02 | 23.30 | 1.87 | 0.971 | Zinc finger CCCH-type containing 12A (ZC3H12A), mRNA |
| 102265044 | 192.19 | 52.78 | 1.86 | 0.944 | Myosin, light chain 9, regulatory (MYL9), transcript variant X2, mRNA |
| 102273821 | 6.90 | 1.90 | 1.86 | 0.897 | Heat shock 70kDa protein 6 (HSP70B') (HSPA6), mRNA |

GRP: growth-retarded yaks pasturing. GNP: growth normal yaks pasturing. DEGs was defined as fold changes ≥ 2 (log2Ratio(GRP/ GNP) ≥1) and probability ≥ 0.8. The FPKM is fragments per kilobase of transcript per million fragments mapped, which represents the expression level of genes.

**Supplementary Table 3.** The top 20% downregulated DEGs in ruminal epithelium of growth-retarded yaks compared to normal yaks. (Based on the fold-change)

| Genes ID | GRP average FPKM | GNP average FPKM | log2Ratio  (GRP/ GNP) | Probability | genes |
| --- | --- | --- | --- | --- | --- |
| 102277315 | 0.01 | 4.11 | -8.68 | 1.000 | Heterogeneous nuclear ribonucleoprotein C-like (LOC102277315), partial mRNA |
| 102280382 | 0.01 | 1.11 | -6.79 | 1.000 | Guanylate-binding protein 4 (LOC102280382), partial mRNA |
| 102271342 | 16.25 | 138.86 | -3.09 | 0.973 | 60S ribosomal protein L27a pseudogene (LOC102271342), misc_RNA |
| 102272391 | 0.49 | 3.02 | -2.63 | 0.861 | Ureidoimidazoline (2-oxo-4-hydroxy-4-carboxy-5-) decarboxylase (URAD), mRNA |
| 102265278 | 11.05 | 65.63 | -2.57 | 0.975 | Keratin 7, type II (KRT7), mRNA |
| 102279576 | 0.35 | 1.92 | -2.47 | 0.901 | Chromosome unknown open reading frame, human C4orf19 (LOC102279576), mRNA |
| 102266457 | 1.05 | 5.59 | -2.42 | 0.850 | Anterior gradient 3, protein disulphide isomerase family member (AGR3), mRNA |
| 102268462 | 1.33 | 6.66 | -2.33 | 0.987 | Acyl-coenzyme A amino acid N-acyltransferase 2 (LOC102268462), mRNA |

GRP: growth-retarded yaks pasturing. GNP: growth normal yaks pasturing. DEGs was defined as fold changes ≥ 2 (log2Ratio(GRP/ GNP)≤-1) and probability ≥ 0.8. The FPKM is fragments per kilobase of transcript per million fragments mapped, which represents the expression level of genes.

**Supplementary Table 4.** Information of the 16S rRNA gene sequencing

| Samples | Raw reads | Effective reads | Taxon reads | OUT number |
| --- | --- | --- | --- | --- |
| GRP 1 | 78,501 | 65201 | 57917 | 1784 |
| GRP 2 | 83,760 | 68619 | 56267 | 1742 |
| GRP 3 | 77,486 | 63468 | 57840 | 1998 |
| GRP 4 | 87,676 | 72406 | 63244 | 1876 |
| GRB 1 | 89,525 | 74141 | 64750 | 1825 |
| GRB 2 | 73,185 | 60614 | 55079 | 1822 |
| GRB 3 | 79,879 | 67172 | 63099 | 1673 |
| GRB 4 | 87,870 | 73779 | 68119 | 1808 |
| GRBC 1 | 88,490 | 74819 | 69652 | 1644 |
| GRBC 2 | 74,099 | 61284 | 57273 | 1566 |
| GRBC 3 | 84,446 | 71033 | 66649 | 1545 |
| GRBC 4 | 87,947 | 74381 | 70372 | 1722 |
| GRBY 1 | 86,071 | 70617 | 63728 | 1778 |
| GRBY 2 | 84,536 | 70005 | 62046 | 1647 |
| GRBY 3 | 37,490 | 30420 | 27320 | 1789 |
| GRBY 4 | 76,331 | 62752 | 55210 | 1710 |
| GNP 1 | 66,249 | 54126 | 44741 | 1651 |
| GNP 2 | 84,238 | 69961 | 64501 | 1906 |
| GNP 3 | 72,976 | 62136 | 58434 | 1776 |
| GNP 4 | 77,239 | 64382 | 59597 | 1716 |

GRP: growth-retarded yaks pasturing. GRB: growth-retarded yaks feeding basal ration, GRBC: growth-retarded yaks feeding basal ration addition CSH. GRBY: growth-retarded yaks feeding basal ration addition ADY. GNP: growth normal yaks pasturing.

**Supplementary Table 5.** Effects of nutritional interventions on Chao 1 and Shannon index of rumen bacteria.

| Items | Groups | | | | | SEM | *P*-Value |
| --- | --- | --- | --- | --- | --- | --- | --- |
|  | GRP | GRB | GRBC | GRBY | GNP |  |  |
| Chao1 | 1799.82 ^a^ | 1686.43 ^b^ | 1560.70 ^c^ | 1647.78 ^bc^ | 1699.93 ^b^ | 22.104 | 0.002 |
| Shannon | 8.04 ^b^ | 8.09 ^ab^ | 7.80 ^c^ | 8.22 ^ab^ | 8.28 ^a^ | 0.048 | 0.004 |

GRP: growth-retarded yaks pasturing. GRB: growth-retarded yaks feeding basal ration, GRBC: growth-retarded yaks feeding basal ration addition CSH. GRBY: growth-retarded yaks feeding basal ration addition ADY. GNP: growth normal yaks pasturing. Data with different small letter superscripts within the same row are significantly different (*P* < 0.05). SEM: standard error of the mean.
